# A Telomerase Immortalized Human Proximal Tubule Cell Line with a Truncation Mutation (Q4004X) in Polycystin-1

**DOI:** 10.1371/journal.pone.0055191

**Published:** 2013-01-28

**Authors:** Brittney-Shea Herbert, Brenda R. Grimes, Wei Min Xu, Michael Werner, Christopher Ward, Sandro Rossetti, Peter Harris, Elsa Bello-Reuss, Heather H. Ward, Caroline Miller, Vincent H. Gattone, Carrie L. Phillips, Angela Wandinger-Ness, Robert L. Bacallao

**Affiliations:** 1 Department of Medical and Molecular Genetics, Indiana University, Indianapolis, Indiana, United States of America; 2 Division of Nephrology, Mayo Clinic, Rochester, Minnesota, United States of America; 3 Department of Anatomy and Cell Biology, Indiana University, Indianapolis, Indiana, United States of America; 4 Department of Pathology, University of New Mexico, Albuquerque, New Mexico, United States of America; 5 Department of Pathology, Indiana University, Indianapolis, Indiana, United States of America; 6 Division of Nephrology and Hypertension, Texas Tech University, School of Medicine, Texas Tech University Health Science Center, Lubbock, Texas, United States of America; 7 Division of Nephrology, Richard L Roudebush VAMC and Indiana University, Indianapolis, Indiana, United States of America; University of Geneva, Switzerland

## Abstract

Autosomal dominant polycystic kidney disease (ADPKD) is associated with a variety of cellular phenotypes in renal epithelial cells. Cystic epithelia are secretory as opposed to absorptive, have higher proliferation rates in cell culture and have some characteristics of epithelial to mesenchymal transitions [1], [2]. In this communication we describe a telomerase immortalized cell line that expresses proximal tubule markers and is derived from renal cysts of an ADPKD kidney. These cells have a single detectable truncating mutation (Q4004X) in polycystin-1. These cells make normal appearing but shorter cilia and fail to assemble polycystin-1 in the cilia, and less uncleaved polycystin-1 in membrane fractions. This cell line has been maintained in continuous passage for over 35 passages without going into senescence. Nephron segment specific markers suggest a proximal tubule origin for these cells and the cell line will be useful to study mechanistic details of cyst formation in proximal tubule cells.

## Introduction

Autosomal dominant polycystic kidney disease (ADPKD) accounts for ten percent of the dialysis population in the United States. The disease is characterized by numerous fluid filled cysts lined by a monolayer of epithelial cells. Mutations in PKD1 or PKD2 loci are responsible for most cases of adult polycystic kidney disease [3]. The genes code for polycystin-1 and 2 respectively and the two proteins interact via c-terminal domains [4]. Polycystin-1 is a multifunctional protein with motifs that mediate cell-cell interactions, cell-matrix attachments and the intracellular C-terminus has been shown to have transcription factor activity [3], [5], [6], [7]. Polycystin-2 is also called Trpp2, a calcium channel that is a member of the Trpp channel family [8]. It forms a complex with polycystin-1 and has been shown to mediate calcium signaling upon mechanical stimulation of monocilia [9], [10]. In a prior communication, another cell line with a point mutation in a transmembrane domain of polycystin-1 (ΔL2433) resulted in a lack of flow sensitive [Ca^+2^]_i_ signaling [11]. These cells have normal levels of polycystin-1 but it fails to assemble in primary cilia [11].

In this communication, we describe an immortalized cystic epithelial cell line with a truncation mutation (Q4004X) in the PKD1 locus and this cell line was selected for proximal tubule markers. In addition, a cell line from an age-matched normal kidney was also created. Most ADPKD cell lines appear to have been derived cysts originating from collecting ducts. In the current study, we isolated cyst epithelial cells from an ADPKD kidney, immortalized the cells using telomerase and generated a stable cystic cell line that expresses proximal tubule markers. Identification of the PKD1 mutation revealed a germline Q4004X mutation. We have not identified possible somatic mutations. In addition to the ADPKD cell line, we also generated a cell line from an age-matched normal kidney. Both cell lines were immortalized with human telomerase and maintained in continuous passage for over 35 passages. Both the normal kidney cell line and the ADPKD cell line (PKD Q4004X) express some proximal tubule markers and have primary cilia. The cyst derived proximal tubule cell line expresses similar levels of polycystin-1 as compared to the normal proximal tubule cells. However, there is less uncleaved polycystin-1 in the cystic epithelial cells as compared to the normal human proximal tubule cell line and immuno gold decoration studies confirm that polycystin-1 fails to assemble in the cilia of the PKD cell line. Finally, polycystin-2 is over-expressed in the PKD cell line as compared to the normal proximal cells. The cystic epithelial cells form cysts in 3D Matrigel cultures while normal kidney cells do not. The immortalized ADPKD Q4004X cell line will be a useful tool for the study of proximal tubule cyst formation and comparative studies of its age and sex matched normal kidney cell line.

## Materials and Methods

### Generation of an Immortalized Age and Sex Matched Normal Kidney (NHPTK) and ADPKD Cell Lines

All animal and human studies including the acquisition of human pathological samples to generate cell lines and recombinant DNA work were approved by the Indiana University IACUC (Institutional Animal Care and Use Committee), IBC (Institutional Biosafety Committee) and IRB (Institutional Review Board) respectively. No consent was obtained from human subjects because all tissue samples, from which the cell lines were generated, were pathological samples that arrived in the laboratory after pathology inspection. No identifying information was collected other than age and sex of the patient from which the sample was obtained. This protocol was approved under an Expedited Review process administered by the Indiana University IRB. PKD Q4004X cells were derived from an end-stage polycystic kidney of a 57 year old male undergoing nephrectomy for a renal transplant. Cells were isolated from cysts as previously described [12]. To ensure that epithelial cells were derived from cysts, cysts were dissected from the surface of the polycystic kidney [13]. NHPTK cells were isolated from a 53 year old male whose kidney was deemed unsuitable for transplantation. Cells were maintained in culture using renal epithelia growth (REGM) media (Lonza Walkersville, Walkersville, MD) supplemented with 2% fetal calf serum and grown in 5% CO_2_ atmosphere at 37°C. Tissue procurement was approved by the Institutional Review Board (IRB) at Indiana University School of Medicine under an expedited review (study number 0911-70). All cells were isolated from kidneys without any identifying information other than age and sex data.

### Transduction of PKD Cells with Retroviral hTERT

Primary cell lines isolated as described above were infected with retroviral hTERT (or an empty vector) as previously described [14], [15]. Briefly, amphotrophic PA317 retroviral packaging cells (a generous gift to BH from Dr. Jerry Shay, UT-Southwestern) containing either an empty vector (pLXSN) or hTERT were propagated to collect supernatants [16]. Supernatants containing released amphotropic retroviruses produced from confluent dishes were filtered (pore size, 0.45 µm) and used to infect the kidney cells. Infected cells were selected with 1 µg/ml G418 for two passages and then maintained for at least 35 passages. A paired uninfected primary cell line entered into senescence and failed to grow any further at passage 6. Immortalized cells were analyzed for hTERT expression and activity. In addition, a separate set of NHPTK and PKD cells were also transduced with pBabepuro or hTERT under a puromycin selection marker as described above. The second set of cell lines was created to give researchers a choice of selection markers in future experiments.

### Telomeric Repeat Amplification Protocol (TRAP)

Telomerase activity from cell extracts was analyzed by the TRAP assay with the TRAP-eze Telomerase Detection kit (Serologicals/Invitrogen) and established protocols [14]–[15]. Following PCR amplification of the in vitro TRAP reaction products, the PCR products were run on a 10% non-denaturing acrylamide gel. The gel was exposed without drying to a phosphor screen and visualized on a Phosphor Imager using ImageQuant software (Molecular Dynamics, Sunnyvale, CA). Telomerase activity was estimated as the presence of a 6-bp telomerase-specific ladder; an internal standard PCR control was also included as the product of separate primers and is represented as a 36-bp band. Five hundred cell equivalents of an H1299 lung cancer cell extract served as a positive control for the TRAP assay; lysis buffer only served as a negative control.

### Immune Blot Analysis of Exogenous hTERT Levels

Whole cell lysates were prepared from logarithmically growing cells using 2% sodium dodecyl sulfate (SDS) in 50 mM Tris-HCl. Total protein concentration was determined using the BCA assay (Pierce, Rockford, IL) according to manufacturer’s instructions. Fifty micrograms of each sample were electrophoresed on a 10% SDS polyacrylamide gel and transferred to a PVDF membrane (Amersh, Arlington Heights, IL). The blots were incubated with monoclonal antibodies to hTERT (IA4 antibody, a generous gift from Geron Corporation) followed by anti-mouse IgG secondary antibody coupled to horseradish peroxidase (Jackson ImmunoResearch, West Grove, PA). After washing, the blots were exposed to X-ray film (Kodak, Rochester, NY) using a chemiluminescent substrate (Super Signal, Thermo Pierce, Rockford, IL).

### Mutation Analysis

Analysis of the mutation(s) in the ADPKD cell line was performed by the Molecular Genetics and Proteomics Core at the Mayo Translational PKD Center. Genomic DNA of both cell lines was extracted from a cell pellet following the salting-out method, as previously described [17]. Genomic DNA was PCR amplified for all the coding exons of the *PKD1* and *PKD2* genes, and the corresponding amplicons directly sequenced on both strands following previously published protocols [17], [18], [19], [20]. Briefly, the *PKD2* gene and the single copy part of *PKD1* were amplified from genomic DNA by standard PCR. The duplicated region of *PKD1* was amplified as five, 3 to 9 Kb Long Range PCR (LR-PCR) fragments, by use of primers that are either anchored in the single copy DNA portion or mismatched with the Homologous Genes sequence. LR-PCR fragments were amplified using the rTth DNA polymerase (PE Applied Biosystems, Foster City, CA) in the supplied DMSO containing buffer. Mutations were typically confirmed in a second independent amplification.

### Fluorescence Activated Cell Sorting

FACS sorting was performed on cells labeled with fluorescein conjugated lotus tetraglobinus lectin (LTL) and rhodamine conjugated dolicous biflorus agglutinin (DBA) purchased from Vector Laboratories (Burlingame, CA). Cell were passaged as described above and suspended in phosphate buffered saline supplemented with 1 mg/ml fluorescein conjugated LTL for 30 minutes and 1 mg/ml rhodamine conjugated DBA. The cells were washed three times with phosphate buffered saline and sorted with a BD FACStar Plus Cell Sorter (BD Biosciences, San Jose, CA). Isolated cells were then grown under the conditions described above.

### Cell Cycle Analysis

Cell cycle analysis was performed as previously described [21]. Cells were passaged and suspended in phosphate buffered saline with 1 mM propidium iodide. DNA content was analyzed with a BD FACscan Flow Cytometer (BD Biosciences, San Jose, CA).

#### Population doubling analysis

Growth curves were calculated for normal kidney and PKDQ4004X based on split ratios for passaging. Both telomerase immortalized cell lines are passaged with a split of 1∶6 flasks twice a week. Population doubling gain was calculated as the log(cell number/number of cells plated)/log2. Cells transfected with plasmids lacking a telomerase insert routinely reached senescence by passage.

### Measurement of Transepithelial Resistance

Both telomerase immortalized normal and ADPKD proximal cell lines selected with Lotus tetraglobinus were plated on 12 mm diameter Costar Clear membrane filter supports and grown to confluence for 5 days in the above described growth media (Corning Costar, Cole Parmer, Vernon Hills, IL). Transepithelial resistance was measured using an Epithelial Volt Ohm Meter with chopstick electrodes (EVOM, World Precision Instruments, Sarasota, FL). Each sample was measured in triplicate and measurements were adjusted to a blank filter control. Measured resistance was adjusted to account for the membrane surface area.

### Antibodies and Reagents

Antibodies to NCB1 were generously supplied by I. Kurtz (UCLA, Los Angeles, CA). NM002 and NM005 antiserum raised against the 3^rd^ cytoplasmic loop domain and the last 200 amino acids from the c-terminal of polycystin-1 respectively, were supplied by Angela Wandinger-Ness (University of New Mexico) [22], [23]. 7E12 monoclonal antibody raised against the LRR region of polycystin-1 was provided by Christopher Ward [24]. Other antibodies were purchased from commercial sources including; anti-NHE-1 (BD Biosciences, San Jose, CA), anti-ZO-1 (Hybridoma Bank, University of Iowa, Iowa City, IA), monoclonal anti-actin (Millipore, Billerica, MA), anti-cytokeratin, anti-vimentin (Sigma-Aldrich, St. Louis, MO) and anti-aquaporin 1 (Millipore, Temmecula, Ca). All chemical supplies and buffers were of reagent grade purchased from Thermo Fisher Scientific (Waltham, MA). Human recombinant epidermal growth factor (EGF) was purchased from Teva Pharmaceuticals (Teva, Israel). 8-bromo-cyclic adenosine monophosphate (8-Br-cAMP) was purchased from Sigma-Aldrich (St. Louis, MO).

### Membrane Preparation

Cells were grown to confluence on 150 mm plates and washed with ice cold phosphate buffered saline. Cells were scraped in ice cold phosphate buffered saline supplemented with protease inhibitor cocktail (Sigma, St. Louis, MO). After centrifuging at 14,000 rpm at 4°C for five minutes, the pellet was resuspended in 0.25 M sucrose, 10 mM Tris-Cl, pH 7.5, 0.2 mM CaCl_2_ with protease inhibitors (Sigma, St. Louis, MO). Cell suspensions were lysed by passing eight times through a Balch homogenizer cooled to 4°C. Homogenates were diluted with 5X volumes of 0.25 M sucrose, 10 mM Tris-Cl, pH 7.5, 1 mM EDTA supplemented with protease inhibitors. The resultant suspension was centrifuged at 4000g for five minutes at 4°C and transferred to a sucrose cushion (1.02 M sucrose, 10 mM Tris-Cl, pH 7.5, 1 mM EDTA with protease inhibitors) and centrifuged at 30,000g for 30 minutes at 4°C in a TLS 55 rotor (Beckman, Pasadena, Ca). Cloudy material observed at the interface of the sucrose cushion was collected and centrifuged at 100,000g at 4°C for 45 minutes. Membrane pellets were resuspended in 0.25 M sucrose, 10 mM Tris-Cl, pH 7.5 supplemented with protease inhibitors. Membrane fractions were assayed for protein concentration with BCA Protein Assay (Thermo Fisher Scientific, Rockford, IL).

### Isolation of Exosomes

Growth media containing only 1% bovine serum albumin as a supplement was incubated with either PKD Q4004X or NHPTK cells for 24 hours. Conditioned media was treated with protease inhibitor cocktail tablets (Roche Diagnostics, Mannheim, Germany). Similarly, first void human urine was collected and treated with protease inhibitor cocktail tablets. Exosomes were isolated as described by Gonzales et al. and Hogan et al. [25], [26]. Purified exosomes were resuspended with phosphate buffered saline and the protein concentration of the suspension was determined using a BCA Protein Assay (bicinchoninic assay, Thermo Fisher Scientific, Rockford, IL) using bovine serum albumin as a protein standard for the standard curve. Exosomes were snap frozen in liquid nitrogen and stored at −80°C until used for immune blot analysis.

### Immunoblot and Immunofluorescence Studies

Serial dilutions were performed using all primary antibodies when used for either immunostaining or immune blotting to determine appropriate dilutions. Equivalent amounts of protein were loaded onto SDS-polyacrylamide gels. For blots in which polycystin-1 was to be studied, samples were loaded on 4–12% gradient gels (Invitrogen, Carlsbad, CA) and run at 25 volts for 16 hours. Proteins were transferred to nitrocellulose at 25 volts for 4 hours at 4°C in 10 mM caps, pH 11.0, 0.01% SDS with 10% methanol. All membranes were blocked with 3% calf serum dissolved in tris buffered saline.

To analyze polycystin-1 expression in exosomes, 40 µg of total protein was loaded per lane in a 3–8% gradient polyacrylamide gel (Invitrogen, Carlsbad, CA) and the gel was run at 150 V for 90 minutes. High molecular weight protein standards (HiMark, Invitrogen, Carlsbad, Ca) were loaded on the gels to assess transfer and relative molecular weights. After running the samples, gels were soaked in 2X transfer buffer with 0.02% SDS for 10 minutes. Transfer to nitrocellulose was performed in a tris-glycine buffer with 0.01% SDS running the transfer at 24 V for 1 hour. After blocking the blot with 1% non-fat dried milk (BioRad, Hercules, CA) dissolved in tris-buffered saline with 0.1% Tween-20, the blot was developed with monoclonal anti-polycystin-1 (7E12) and rabbit anti-mouse IgG1 (Southern Biotechnology, Birmingham, AL). Blots were visualized with Super Signal (Thermo Fisher Scientific, Rockford, IL).

To perform immune-staining studies, cells were grown on glass cover slips and fixed with 2% paraformaldehyde dissolved in phosphate buffered saline for 10 minutes. Fixation reactions were quenched with 100 mM NH_4_Cl dissolved in phosphate buffer saline. Samples were permeabilized with 2% saponin dissolved in Tris-buffered saline (TBS) or with TBS with 0.1% Triton X-100.

### Transmission Electron microscopy

Cells were grown on Thermanox cover slips (NUNC, Thermo Fisher Scientific, Waltham, MA). After 10 days post plating they were fixed with 4% Paraformaldehyde in 0.1 M phosphate buffer. After fixation and rinsing in buffer pre-embedding immunostaining was done. Cover slips were immersed in the primary antibody overnight at 4°C, rinsed the next day in buffer and placed in the secondary antibody attached to 10 nm colloidal gold (AURION, Hatfield, PA) for 2 hours. After several rinses in buffer the samples were dehydrated through a graded series of ethyl alcohols and embedded in Embed 812 (Electron Microscopy Sciences, Hatfield, PA). Thin sections (70–90 nm) were cut, dried on grids and stained for contrast using uranyl acetate. The grids were viewed with a Tecnai G 12 Bio Twin transmission electron microscope (FEI, Hillsboro, OR) and images taken with an AMT (Advanced Microscopy Techniques, Danvers, MA) CCD camera.

### Scanning Electron Microscopy

Cell cultures grown on Thermanox coverslips (Nalge Nunc International, Rochester, NY) were fixed with 2% paraformaldehyde/2% glutaraldehyde/0.1 M phosphate buffer. After initial fixation, the specimens were rinsed with PBS (phosphate buffered saline) followed by post fixation with 1% osmium tetroxide/0.1 M phosphate buffer for 2 hours. After rinsing with PBS the specimens were dehydrated using a series of graded ethyl alcohols from 70%–100%. The specimens were then critical point dried (Samdri-790, Tousimi, Rockville, MD). After drying, the specimens were mounted on aluminum stubs with adhesive tabs and sputtered coated for 3 minutes with gold/palladium (Polaron, Energy Beam Sciences, Agawam, MA). The specimens were then viewed and images taken with a JEOL 6390LV (Peabody, MA) scanning electron microscope in SEI imaging mode, 5 KV, 12 mm working distance, and spot size 30.

### Measurement of Cell Volume

Both cell lines were grown to confluence and then passaged with trypsin-EDTA solution to create cell suspensions with a density of 100,000 cells/ml. Cell volume was measured with a Beckman Coulter Counter (Miami, FL). Twelve samples from each cell line were analyzed.

### Growth in 3D Culture

Cells (PKD Q4004X and NHPTK) were added to growth factor reduced Matrigel (GFR Matrigel, B&D Biosciences, Bedford, MA) or type I collagen (Wako Chemical Co, Osaka, JP) at a concentration of 3000 cells per ml and place in glass bottom plastic culture dishes (Mat Tek Corp, Ashland, MA) or in 96 well plates. Cultures were overlaid with culture media and incubated at 37°C in a 5% CO_2_ incubator. Culture media was replaced daily to ensure adequate growth conditions. After cyst formation occurred, usually after 14 days in culture, forskolin (5 µM) or forskolin plus 50 ng/ml IGF-1 was added to the culture to stimulate cyst expansion.

#### Imaging of 3D cultures

Cell and matrix samples were fixed in 4% paraformaldehyde dissolved in phosphate buffered saline for 30 minutes at room temperature. Fixation reactions were quenched by incubating the samples with 100 mM NH_4_Cl dissolved in phosphate buffered saline for 30 minutes. After several washes with phosphate buffered saline, samples were permeabilized with 0.1% Triton X-100 dissolved in phosphate buffered saline. Samples were labeled with Bodipy phalloidin (Life Technologies, Carlsbad, CA) and Hoechst 33342, washed with 0.1% Triton X-100, phosphate buffered saline, pH 7.4 and post-fixed in 2% paraformaldehyde, phosphate buffered saline. Matrix samples were removed from the wells and placed on glass bottom plastic culture dishes (Mat Tek Corp, Ashland, MA). Confocal images were collected as previously described using an Olympus Flowview confocal microscope equipped for two photon confocal microscopy [27].

## Results

**Figure 1:**
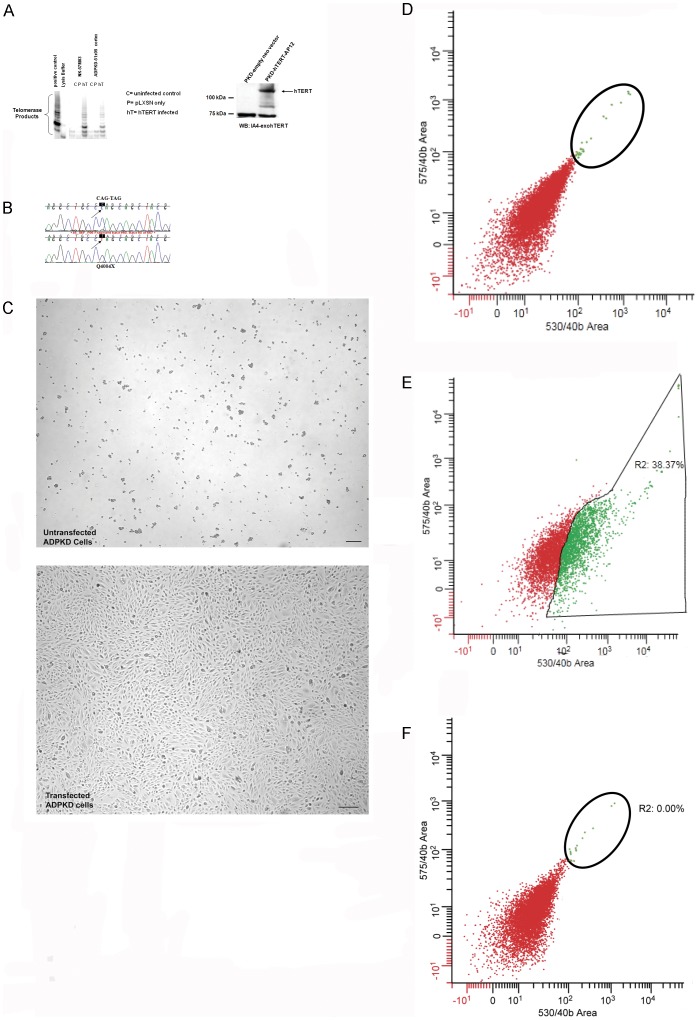
Characterization of PKD Q4004X growth and selection. A: Telomerase activity assay and immune blot analysis of transduced and untransduced cells. Left panel: Telomerase activity detected as labeled telomerase product telomeric DNA ladders. DNA ladders are only observed in cells transduced with hTERT. Untransduced control cells or cells transduced with empty vector (pLXSN) have no detectable telomerase activity. Right panel: Exogenous telomerase expression confirmed by immune blot analysis. A 120 kDa band is observed in lysates made from PKD cells transfected with hTERT. **B:** **Mutation detection in the PKD cell line.** A C to T mutation resulting in a premature truncation at amino acid 4004 is shown. **C:** Phase contrast image of untransduced cells (upper image) and hTERT transfected PKD cells (lower image) maintained in selection media. **D:** **Fluorescence activated cell sorting of LTL and DBA labeled HK2 cells.** Fluorescence intensity at 575 nM/unit cell area versus fluorescence intensity at 530 nM/unit cell area are plotted on the Y and X axis respectively. Since DBA was tagged with rhodamine, cells labeled with the DBA marker are expected to register in the region demarcated by the circle. **E:** **Fluorescence activated cell sorting of LTL and DBA labeled MDCK-II cells.** Fluorescence intensity at 575 nM/unit cell area versus fluorescence intensity at 530 nM/unit cell area are plotted on the Y and X axis respectively. Since DBA was tagged with rhodamine, cells labeled with the DBA marker are expected to register in the region demarcated by the circle and shown in green. MDCK-II cells are a mixed population basted on this assay (compare red and green populations). **F:** **Fluorescence activated cell sorting of LTL and DBA labeled PKD Q4004X cells.** Fluorescence intensity at 575 nM/unit cell area versus fluorescence intensity at 530 nM/unit cell area are plotted on the Y and X axis respectively. Since DBA was tagged with rhodamine, cells labeled with the DBA marker are expected to register in the region demarcated by the circle. Few cells have DBA labeling with greater than 99% of the cells showing LTL positive labeling.

Primary cultured renal epithelial cell lines were developed from pooled dissected cysts as previously described [28]. These cells were obtained from a male in the fifth decade of life that had a diagnosis of ADPKD. An age and sex matched primary culture cell line isolated from a normal kidney was immortalized along with the PKD cell line for comparison. Both primary cell lines were sensitive to neomycin selection and following transduction were neomycin resistant (Figure 1C and data not shown). We also found that immortalization was successful without selection because under our culture conditions, untransduced cells failed to grow after passage 6. Growth failure at this passage is probably due to senescence. Both western blot analysis and telomerase activity assays confirmed that the cell lines over-expressed human telomerase (Figure 1A and data not shown). Sequence analysis of the PKD1 loci in the cystic cell line revealed a premature stop codon that would result in a truncation of polycystin-1 at position Q4004X (Figure 1B). This would eliminate the last 299 amino acids from the carboxy terminal. No second mutation was detected and there were no mutations detected in the PKD2 locus (data not shown). Notably the height of the peak at position Q4004 (C→T, red peak) is equivalent to the C peak (blue), suggesting that the mRNA bearing the mutation is expressed at roughly equivalent levels as the wild-type message (Figure 1B and data not shown).

Untransfected cells failed to grow in selection media (Figure 1C, upper panel and data not shown) while the transfected cells formed confluent monolayers in the selection media (Figure 1C, lower panel).

Once the cells passed the point when we typically observed senescence, we selected for expression of a proximal tubule marker by performing FACs sorting on both normal and cystic epithelial cell lines. After passaging the cells, fluorescein tagged lotus tetraglobinus lectin (LTL) and rhodamine conjugated dolichous biflourus was used to label cells in suspension. Control experiments were performed using HK-2 cells and MDCK-II cells, a human proximal tubule cells line and a mixed population cell line respectively. The results of the fluorescence sort are shown in figure 1 (D, E, and F). HK-2 cells had a fluorescent signature comprised of predominantly equivalent signals from the FITC and Rhodamine channels (Figure 1D). A small percentage of cells had a high intensity rhodamine signal (Figure 1D, green dots in the circled area). In contrast MDCK-II cells had a shifted fluorescent signature (Figure 1E). The PKD Q4004X cell line has a fluorescent lectin binding pattern most similar to the HK cells (Figure 1F compare to 1D). Cells binding the LTL lectin were collected and maintained in culture.

**Figure 2:**
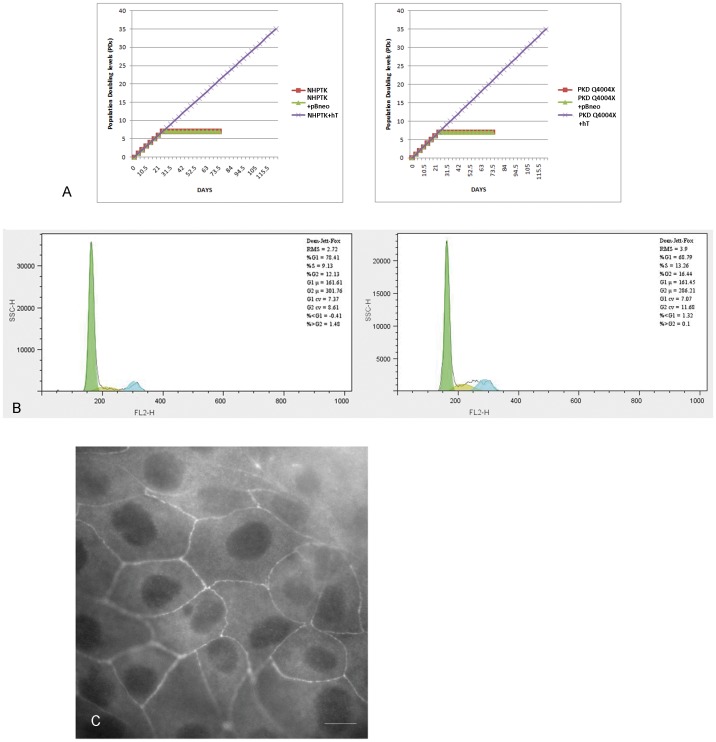
Cell Growth Characteristics of NHPTK and PKD Q4004X Cells. A: Population doublings of normal and cystic proximal tubule cell lines. Graphical depiction of population doublings based on growth and passage number. Left panel NHPTK cell line population doubling, right panel PKD Q4004X cell line population doubling**. B: Cell cycle analysis of telomerase immortalized PKD Q4004X and NHPTK cell lines.** Left panel PKD Q4004X cells labeled with propidium iodide. Right panel NHPTK cells labeled with propidium iodide. Cells were passaged as described in materials and methods. Representative results are shown from 3 separate experiments. **C: ZO-1 expression in PKD Q4004X cells.** ZO-1 is expressed at cell-cell borders typical of its expression pattern in renal epithelial cells.

Figure 2A graphically depicts the population doublings and extended life span of hTERT transduced cell lines for both the normal human proximal tubule cell line (NHPTK) and the Q4004X proximal PKD cell line. Both cell lines were found to have the same doubling rate. However, doubling times increased when either cell line was plated at lower densities (data not shown). The average cell volume of the PKD Q4004X cell line was 11.9% greater than the NHPTK cell line as measured by Coulter counters (83.3 femtoliters versus 79.2 femtoliters, N = 18, p<0.08). This data suggests a trend toward significance but our studies never achieved statistical significance. Cell cycle analysis in non-synchronized cells revealed no significant differences between the two cell lines (Figure 2B). Typically, the percentage of cells in G1 ranged between 68 and 72% in both cell lines (Figure 2B) and the percentage of cell in S phase varied between 8 and 14% while the percentage in G2 fluctuated between 12 and 16% with no clear distinction between the two cell lines (Figure 2B and data not shown).

**Figure 3:**
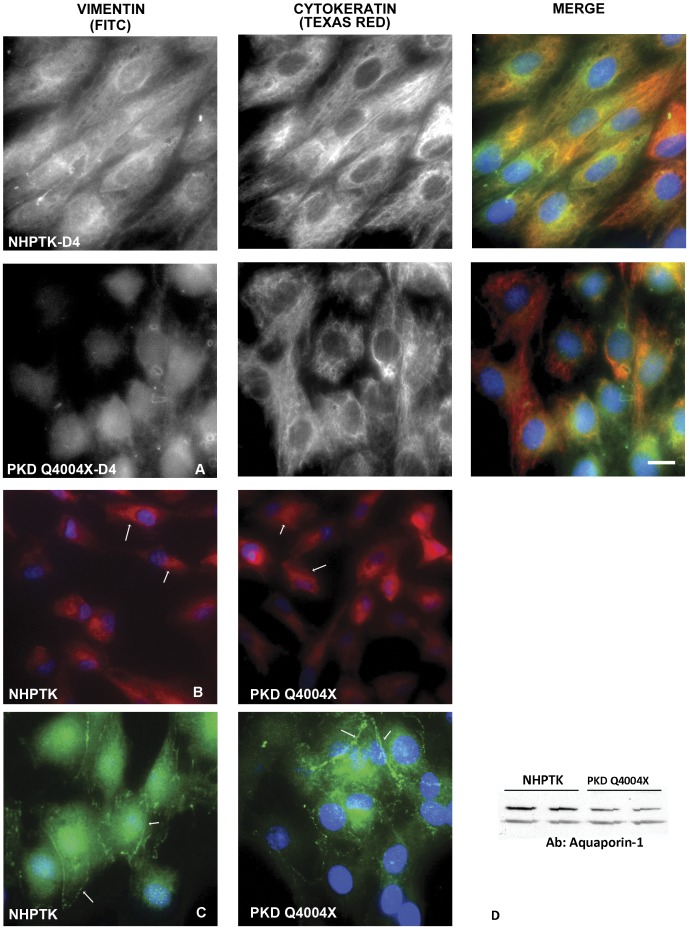
Immunostaining of PKD Q4004X and NHPTK cells. A: Vimentin and cytokeratin staining. Telomerase immortalized PKD Q4404X and NHPTK proximal tubule cells were maintained in culture for four days. After fixation and staining, samples were imaged with a wide-field microscope. Intermediate filament components, vimentin and cytokeratin are observed even after four days in culture. Bar = 10 µm. **B:** **NCB1 staining.** Telomerase immortalized PKD Q4004X and NHPTK proximal tubule cells were maintained in culture for three days. Cells were imaged with a wide-field microscope after fixation and staining. 60X objective. **C: Aquaporin-1 expression.** Immortalized PKD Q4004X and NHPTK proximal tubule cells were maintained in culture for three days. Cells were fixed and stained with anti-aquaporin-1. Images were collected with a wide-field microscope using a 60X oil objective (NA = 1.41).

When grown on filter supports, both normal and PKD cell lines formed low resistance monolayers with trans-epithelial resistance in the range of 40–80 ohms-cm^2^ after adjusting for the background resistance of the filter supports (data not shown). Immunostaining for ZO-1, a tight junction associated protein, showed a continuous ring of staining at the apical region of the cells (Figure 2C). To evaluate other epithelial characteristics we stained cells for vimentin, cytokeratin 24, NHE-3, aquaporin-1 and NCB1. As noted in figure 3, PKD Q4004X cells expressed cytokeratin, NCB1 and aquaporin-1 but did not express NHE-3 (Figure 3 A,B,C and data not shown). Vimentin staining was maintained in both cell lines even after 5 days in culture, suggesting that some component of dedifferentiation or transdifferentiation had occurred (Figure 3A). NCB1 staining revealed expression only in intracellular compartments (Figure 3B, arrows) with no observable membrane assembly of NCB1. In contrast, aquaporin-1 w, as found on the plasma membranes on both NHPTK and PKD Q4004X cells (Figure 3C, arrows). To confirm aquaporin-1 expression in both cell lines we performed an immune blot of lysates from NHPTK and PKD Q4004X cells (Figure 3D). Under our immune blot conditions using a gradient gel we found two bands (29 kDa and ∼33 kDa) in both cells corresponding to monomeric aquaporin-1 and the glycosylated form of aquaporin-1 respectively [29].

**Figure 4:**
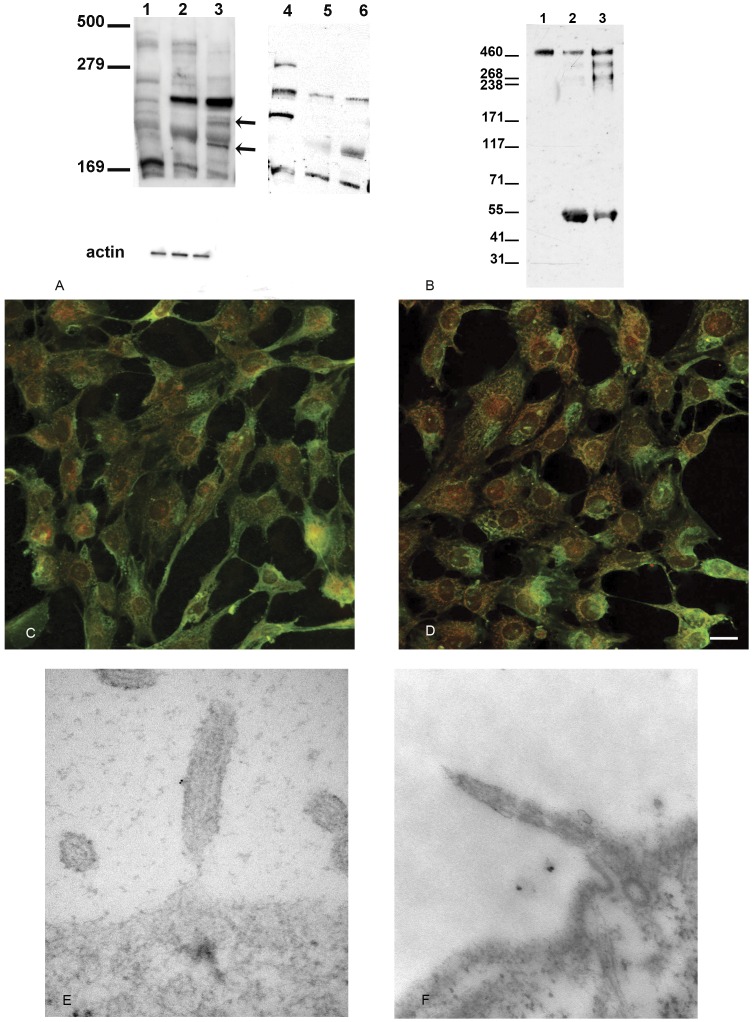
Polycystin-1 expression and staining in RPTEC, NHPTK and PKD Q4004X cells. A: Immune blot of membrane preparations from RPTEC, NHPTK and PKD Q4004X cells. Membranes were isolated as described in materials and methods. Lanes 1, 2 and 3: Immune blot analysis using anti polycystin-1 (NM 005). Lane 1 membrane preparation from renal proximal tubule renal epithelial cells (RPTEC). Lane 2 membrane preparation from telomerase immortalized normal kidney proximal tubule cells. Lane 3 membrane preparation from PKD Q4004X cells. Arrows point to bands that are unique to the PKDQ4004X cells (Lane 3). Lower panel: immune blot from the lower portion of the gel probed with anti-actin antibodies. Similar levels of actin are present in the blot. Lanes 4, 5 and 6: Immune blot analysis using anti-LRR polycystin-1 (7E12) used to probe the same blot after stripping NM005. Lane 4 membrane preparation from RPTEC cells. Lane 5 membrane preparation from telomerase immortalized NHPTK cells. Lane 6 membrane preparation from telomerase immortalized PKD Q4004X cells. **B: Immune blot of exosomes.** Exosomes were isolated as described in materials and methods section. Blot was probed with a monoclonal antibody raised against the leucine rich region of polycystin-1 (7E12). Lane 1 exosomes from human urine. Lane 2 exosomes from NHPTK cells. Lane 3 exosomes from PKD Q4004X cells. Full length polycystin-1 is observed in all sources of exosomes. More truncated polycystin-1 is observed in the exosomes derived from the PKD cell line. **C,D: Immune staining of polycystin-1 using 7E12 and NM 005 in telomerase immortalized kidney cells.** Telomerase immortalized proximal tubule cells (C) or PKD Q4004X (D) were grown to confluence, fixed and stained with monoclonal anti-LRR (7E12) and polyclonal anti polycystin-1 (NM005). The image shown is a merged image in which 7E12 was labeled with Alexa 488 and NM 005 was labeled with rhodamine. Extensive co-localization of both antibodies is observed in which a reticular peri-nuclear structure is labeled. Some areas of the reticulum appear to be labeled with only one of the antibodies. Pearson’s correlation coefficient was 88% for both cell lines (N = 3). Bar = 10 µm. **E, F: Immuno-gold decoration of cilia.** Telomerase immortalized proximal tubule (E) and PKD Q4004X (F) cells were grown to confluence and processed for immune gold decoration using anti polycystin-1 (NM 005). Gold decoration of cilia was only observed in the normal kidney cells (E). None of the cilia observed in PKD cells were positive for immune gold staining (F).

Given the results of the PKD1 gene analysis we examined expression of polycystins in the two cell lines to determine what the effect of the truncation mutation has on polycystin-1 biogenesis. Immunoblot analysis of cell lysates made with RIPA buffer failed to show any significant differences in the molecular weight of polycystin-1 using either anti-sera raised against the c-terminal 200 amino acids of polycystin-1 (NM005) or the amino terminal LRR domain (7E12) (data not shown). Immune blots of membrane preparations made from renal proximal tubule epithelial cells (RPTEC), NHPTK cells or PKD cells did show differences in polycystin-1 expression (Figure 4A). NM005 antiserum revealed bands at approximately 480, 260, 248, 200 and 172 kDa in RPTEC and NHPTK cells (Figure 4A, lanes 1 and 2). In the NHPTK cells a strong band at 240 kDA is matched by a similar dominant band in the PKDQ4004X cells (Figure 4 A compare lanes 2 and 3). However little uncleaved polycystin 1 was observed in the membrane preparation derived from PKDQ4004X cells (Figure 4, lane 3) as compared to the levels observed in the RPTEC and NHPTK cell lines (Figure 4, lanes 1,2). Additional bands are also observed in the molecular weight range ∼220 and 195 kDa range. These bands were observed in all repeat studies and we observed in extracts or membrane preparations from the PKDQ4004X line (data not shown). Below the top panel of Figure 4A we show an actin immunoblot from the same experiment demonstrating that the relative amount of protein loaded per well was equivalent. Anti-LRR (7E12) (Figure 4A, lanes 4, 5 and 6) bound to five bands at relative molecular weights of 300, 260, 248, 200 and 172 kDa (Figure 4A, lanes 4, 5 and 6). RPTEC cells strongly express polycystin-1 fragments of 300, 260, 248 and 172 kDa. In contrast NHPTK and PKDQ4004X express bands at 260, 200 and 172 kDa (Figure 4A, Lanes 5 and 6). PKDQ4004X cells express more of the 200 kDa fragment as compared to NHPTK cells. However we did not observe a truncated polycystin-1 fragment in the PKDQ4004X membrane preparations as would be predicted from the mutation analysis. To further evaluate the biogenesis of polycystin-1 in the PKDQ4004X cell line we analyzed polycystin-1 expression in exosomes preparations. As shown in Figure 4B, full-length polycystin-1 was observed in urine exosomes (Figure 4B, lane 1) and by exosomes isolated from both NHPTK and PKD Q4004X cells (Figure 4B, lanes 2–3 respectively). An addition band at ∼55 kDa is also observed in the exosomes fractions obtained from NHPTK and PKD Q4004 cells. These bands are likely IgG heavy chains. In lane 3, two additional high molecular weight bands are also observed at ∼350 and 240 kDa. More of this cleaved form of polycystin-1 is observed in the exosomes isolated from PKD Q4004X cells.

Co-staining cells with NM005 and 7E12 in sub-confluent cells showed no difference in staining when cilia are absent (Figure 4 C, D). The staining pattern is consistent with rough endoplasmic reticulum and Golgi compartment staining. Polycystin-1 was observed along the lateral membrane and a perinuclear membranous compartment (Figure 4 C, D). In both cell lines, NM005 and 7E12 were 87% co-localized. Ultrastructural analysis with NM005 immuno gold labeling showed that primary cilia of the normal kidney cells contained polycystin-1 (Figure 4E). In contrast, none of the primary cilia examined in PKD Q4004X cells expressed polycystin-1 (Figure 4F). Therefore, aside from the lack of cilia staining, there were no other appreciable differences in polycystin-1 localization between the NHPTK and PKD Q4004X cells.

**Figure 5:**
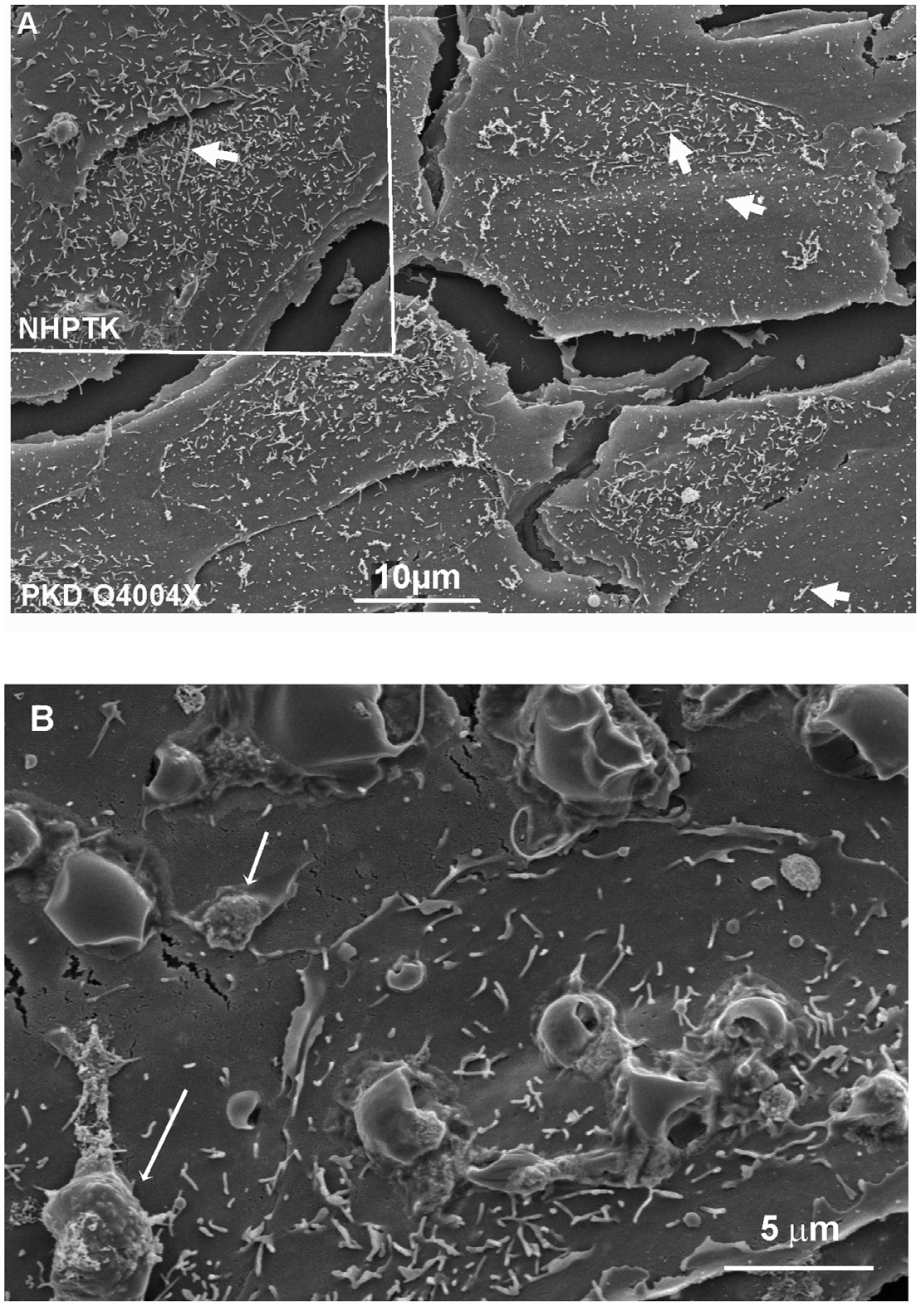
Scanning electron micrographs of NHPTK and PKD Q4004X cells. A: PKDQ4004X cells have sparse microvilli and short monocilia (arrows) as compared to NHPTK (arrow). Scale bar-10 µm is the same for both PKD Q4004X and NHPTK images. B: Apical membrane surface PKD Q4004X at high magnification. Membrane blebs are seen (arrows) which are roughly the size of exosomes. Bar = 5 µm.

Differences in polycystin localization revealed a lack of polycystin-1 in monocilia in the PDK Q4004X line prompted an examination of the cilia in both cell lines. This finding prompted us to examine the two cell lines using scanning electron microscopy. In figure 5 scanning electron photomicrographs are shown with the same scale. NHPTK cells are smaller in size than the PKD Q4004X cells. Furthermore we observed that NHPTK cells have longer cilia (Figure 5 A and B). In addition on the membrane surface of PKD Q4004X cells we saw surface membrane blebs (Figure 5B, arrows) which were not observed on NHPTK cells.

**Figure 6:**
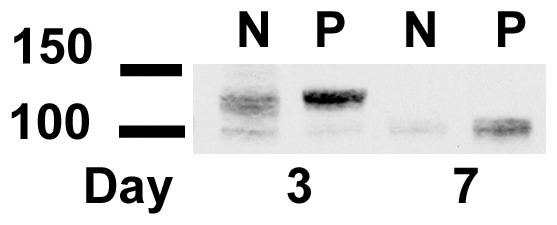
Polycystin-2 expression in telomerase immortalized proximal tubule cells. Both telomerase immortalized normal kidney (N) proximal and PKD Q4004X (P) cells were grown to confluence for 3 or 7 days. Extracts were made with buffers containing non-ionic detergent and equivalent amounts of protein (50 µg) were loaded in each lane. Lanes labeled N and P represent lysates made from telomerase immortalized normal kidney proximal tubule and PKD Q4004X cells, respectively. Two bands at molecular weights 120 and 100 kDa are seen and the PKD cells have higher expression levels at both time points. At 7 days only the 100 kDa band is observed.

To further characterize polycystin expression, we examined polycystin-2 expression in the two cell lines. Immune blot revealed that polycystin-2 is expressed in greater amounts in the PKD Q4004X cell line. In lysates made 3 days after plating at a density of 50,000 cell/cm^2^, a doublet of 130 kDa and a single band at 100 kDa was observed in both cell lines. The 130 kDa isoform is expressed at higher levels in the cystic cell line. In lysates made from cells grown in culture for 7 days, only the 100 kDa band was observed and there is more polycystin-2 expression in the PKD cell line (Figure 6). The observation that two forms of polycystin-2 are expressed in these cells suggests that there are at least two spice variants expressed in these cell lines. A polycystin-2 spice variant with an exon 7 deletion has been noted with a molecular weight of 103 kDa, a size in good agreement with the lower molecular weight band seen in our blot [30]. Immune staining with the same antibody was not robust under a variety of fixation and staining conditions; however weak polycystin-2 staining was observed in an intracellular compartment in both cell lines, consistent with a rough endoplasmic reticulum staining pattern. Cilia staining were not observed in either cell type (data not shown).

**Figure 7:**
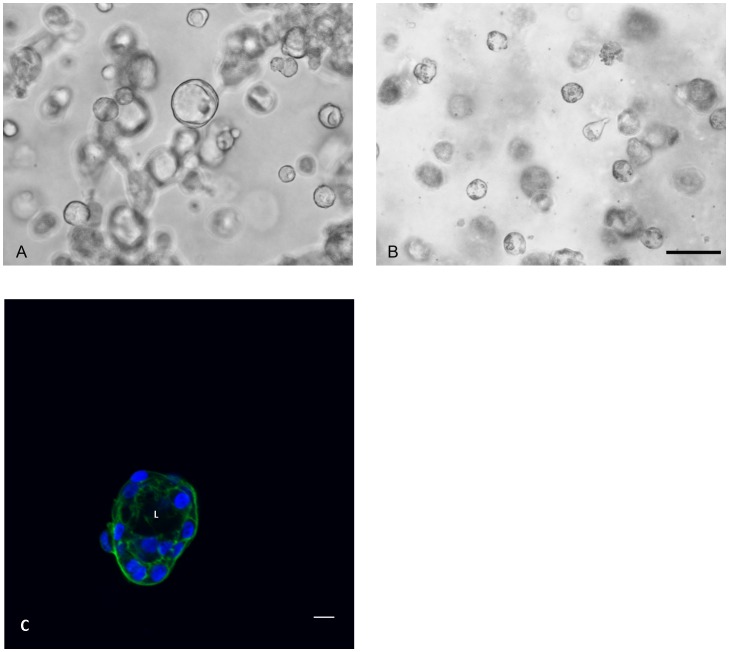
Cyst formation by PKDQ4004X cells grown in Matrigel. Normal and PKDQ4004X cells were plated at a density of 3000 cells per ml of Matrigel with culture media and grown in culture for 14 days. A: Phase contrast image of PKDQ4004X cells forming cysts in Matrigel matrix. B: Phase contrast image of normal kidney cells in Matrigel matrix. Bar = 50 microns. C: Fluorescence photomicrograph of PKD Q4004X cell cyst. Extended focus image of 5 image planes covering a z distance of 5 microns. Actin cytoskeleton is labeled with Bodipy phalloidin (green) and nuclei are labeled with Hoechst 33342 (blue). A clear lumen (L) is surrounded by a layer of epithelial cells to form a cyst. Bar = 10 microns.

Finally, we examined the effect of growing both cell lines in 3D matrix culture. Both PKD and NHPTK cells were plated on collagen matrix at a density of 50,000 cell per cm^2^ and then overlaid with collagen 24 hours after plating. Under identical growth conditions, MDCK cells will form either cysts or tubules depending on the presence of hepatocyte growth factor [31], [32], [33], [34]. The telomerase immortalized cell lines did not form cysts when grown in collagen matrix (data not shown). Both NHPTK and PKD Q4004X cells form tubules when overlaid with type I collagen. However when PKD Q4004X cells were plated with HK-2 cells at a ratio of 1∶10, a few cysts were observed. In contrast plating NHPTK cells with HK-2 cells did not cause cyst formation (data not shown). PKD Q4004X cells formed many cysts when plated in growth factor depleted Matrigel. Cysts were observed within 14 days after plating (Figure 7A, C). Adding forskolin or forskolin in combination with IGF-1 accelerated cyst expansion (data not shown). In contrast NHPTK cells plated under identical conditions never developed cysts or tubule arrays, instead the cells formed small aggregates with intracellular vacuoles observed at cell borders (Figure 7B).

## Discussion

Autosomal dominant polycystic kidney disease is one of the ciliopathies linked to renal cystic disease [35], [36], [37]. In APDKD renal cyst formation can occur in any nephron segment but cystic disease has been suggested to be predominantly arising from distal segments [38]. Based on current evidence it is not clear how mutations in either polycystin-1 or polycystin-2 genes lead to cyst formation. A suggested mechanism proposed is the two-hit hypothesis in which a second somatic mutation is required in the normal allele in a cell that already has a germ line mutation leading to a clonal expansion of cells that have mutations in both alleles [39]. This mechanism readily accounts for the relatively sparse generation of renal cysts.

The cell line developed in this communication was selected by FACs for proximal tubule markers. Although the cells were grown from a mixed population of isolated renal cysts, over 99% of the cells were positive for LTL staining, suggesting that our culture conditions select heavily for cells of proximal origin. It is also possible cysts harvested solely from the surface of ADPKD kidneys are of proximal origin. In four primary cell lines we have analyzed by FACs we found that all of the lines were exclusively LTL positive based on FACs analysis. One of the four cell lines was selected for telomerase immortalization and genotyped based on the fact that this PKD cell line could be paired with an age and sex matched normal kidney cell line for further analysis. Since little is known about the mechanisms of cyst formation by proximal tubule cells the cell lines are a unique resource for the PKD research community.

Telomerase was selected as an immortalization agent because this method was considered less likely to induce secondary effects on growth regulatory pathways that may be affected in the setting of mutations in polycystin-1 or 2 function. Therefore analysis of Myc, Src or Jak/Stat signaling pathways would be less likely to be altered by the immortalization vector. Loghman-Adham et al reported on the creation of cystic epithelial cell lines using SV40 temperature sensitive large T antigen to immortalize the cells. They showed that at the non-permissive temperature, large T antigen was degraded and that epithelial markers were up regulated [40]. However it is not certain that downstream signaling pathways or the pattern of gene expression is truly representative of the de novo mutation in polycystin-1. Since large T antigen expressing transgenic mice have cysts it is likely that large T antigen is cystogenic [41]. Notably SV40 T antigen has been shown to induce polyploidy in cells due to its interaction with Bub1 this would suggest that these cell lines cannot be used to examine genomic instability [42], [43]. In contrast immortalization with human telomerase is, based on our current understanding, less likely to alter growth signaling responses or induces genomic instability [44]. However a few papers have described alterations in a growth regulatory signaling pathway associated with telomerase over-expression [45], [46]. For instance, Li et al., demonstrated up regulation of macrophage colony stimulating factor-1 receptor (CSF1R) message and protein levels in hTERT-immortalized human ovarian surface epithelial cells and in human pancreatic epithelial cells [46]. In hTERT-immortalized human ovarian surface epithelial cells stimulation of CSF1R with colony stimulating factor-1 causes hTERT translocation into the nucleus and c-Myc binding to the hTERT promoter region [46]. SiRNA knockdown of CSF1R blocked these effects and inhibited cell immortalization. This data suggests that hTERT immortalization in renal epithelial cells may be modulated by other growth factor signaling pathways. In other modes of immortalization, such as adenovirus transduction, adenovirus mediated immortalization involves transcriptional activation of hTERT by E1A acting cooperatively with E1B activation of Ras [47].

Analysis of polycystin-2 expression in the PKD Q4004X-LTL cell line as compared to the normal kidney cell lines revealed no differences in sub cellular localization. However more polycystin-2 was noted in PKD Q4404X cells and two polycystin-2 isoforms were expressed in both cell lines. Alternative splicing variants of polycystin-2 has been described in brain and one splice variant lacking exon 7 does not interact with polycystin1 [48]. By comparison, we observed striking differences in polycystin-1 expression in the PKD Q4004X-LTL cells in that there was significantly more uncleaved polycystin-1 present in these cells as determined by immunoblot of membrane fractions. A current hypothesis to explain the focal nature of cyst formation have suggested that two hits are required for cytogenesis: the first mutation being a germ-line transmitted mutation and the second mutation is a spontaneous somatic mutation [49]. Alternatively, but not necessary exclusionary, mutations in polycystin-1 affect flow mediated calcium signaling mediated by mechanical deformation of cilia [50], [51]. In a separate communication, this cell line has a defect in calcium signaling secondary to alterations in purinogenic receptor function [52]. Four differences in polycystin-1 biogenesis are noted in this cell line, first polycystin-1 was not observed in monocilia, second anomalous sized bands were observed in western blots probed with a polyclonal antibody raised against 200 amino acids from the c-terminus of polycystin-1, there is quantitatively more lower molecular weight forms of polycystin-1 observed exosomes isolated from the PKD Q4004X-LTL line and lastly there was less uncleaved full length polycystin-1 observed in the PKD Q4004X-LTL cell line (figure 4A). One potential interpretation of this finding is that the mutant form of polycystin-1 is acting as a dominant negative that prevents cleavage at the GPS site [53] or inhibits assembly into monocilia. Only one allele of polycystin-1 was found to be mutated in this cell line. While this finding is contrary to the prediction of the “two hit” hypothesis, we cannot eliminate the possibility that an undetected point mutation exists in the other polycystin-1 allele [20], [54]. It is also possible that since the cell line was created from a mixed population of cysts, the second hit could not be identified using current mutation detection techniques. Conversely if a second hit does not exist in this cell line and the Q4004X mutation acts as a dominant negative, then the cells are functionally equivalent to cell lines with mutations in both HmPKD1 alleles [53].

In conclusion, we have characterized a polycystic kidney renal epithelial cell line that expresses proximal tubule markers. This cell line appears to have a single truncation mutation at position Q4004X and western blot analysis reveals that the cell line has significant amounts of uncleaved polycystin-1. In three dimensional cultures using type I collagen both cell lines (normal kidney and PKD Q4004X) fail to form either tubules or cysts. But the PKD Q4004X cell line does form cyst when co-cultured with HK cells or when cultured in growth factor reduced Matrigel. This cell line should be useful to further dissect the cellular phenotype associated with mutations in HmPKD1 and factors required to induce cyst formation in 3D culture.
